# iPLA_2_β Contributes to ER Stress-Induced Apoptosis during Myocardial Ischemia/Reperfusion Injury

**DOI:** 10.3390/cells10061446

**Published:** 2021-06-09

**Authors:** Tingting Jin, Jun Lin, Yingchao Gong, Xukun Bi, Shasha Hu, Qingbo Lv, Jiaweng Chen, Xiaoting Li, Jiaqi Chen, Wenbin Zhang, Meihui Wang, Guosheng Fu

**Affiliations:** 1 Department of Cardiology, Sir Run Run Shaw Hospital, Zhejiang University School of Medicine, Hangzhou 310020, China; 11818361@zju.edu.cn (T.J.); 288669@zju.edu.cn (J.L.); 21818353@zju.edu.cn (Y.G.); bixukun@zju.edu.cn (X.B.); hushasha503@163.com (S.H.); lvqingbo@zju.edu.cn (Q.L.); lixiaoting26@zju.edu.cn (X.L.); 2 Key Laboratory of Cardiovascular Intervention and Regenerative Medicine of Zhejiang Province, Sir Run Run Shaw Hospital, Zhejiang University School of Medicine, Hangzhou 310027, China; CJW19951006@163.com (J.C.); jackiechen684@gmail.com (J.C.)

**Keywords:** iPLA_2_β, lysophosphatidylcholine, ER stress, ischemia/reperfusion injury, apoptosis, translocation

## Abstract

Both calcium-independent phospholipase A2 beta (iPLA_2_β) and endoplasmic reticulum (ER) stress regulate important pathophysiological processes including inflammation, calcium homeostasis and apoptosis. However, their roles in ischemic heart disease are poorly understood. Here, we show that the expression of iPLA_2_β is increased during myocardial ischemia/reperfusion (I/R) injury, concomitant with the induction of ER stress and the upregulation of cell death. We further show that the levels of iPLA_2_β in serum collected from acute myocardial infarction (AMI) patients and in samples collected from both in vivo and in vitro I/R injury models are significantly elevated. Further, iPLA_2_β knockout mice and siRNA mediated iPLA_2_β knockdown are employed to evaluate the ER stress and cell apoptosis during I/R injury. Additionally, cell surface protein biotinylation and immunofluorescence assays are used to trace and locate iPLA_2_β. Our data demonstrate the increase of iPLA_2_β augments ER stress and enhances cardiomyocyte apoptosis during I/R injury in vitro and in vivo. Inhibition of iPLA_2_β ameliorates ER stress and decreases cell death. Mechanistically, iPLA_2_β promotes ER stress and apoptosis by translocating to ER upon myocardial I/R injury. Together, our study suggests iPLA_2_β contributes to ER stress-induced apoptosis during myocardial I/R injury, which may serve as a potential therapeutic target against ischemic heart disease.

## 1. Introduction

Cardiovascular disease remains the leading cause of death worldwide, imposing major burdens on our healthcare and socioeconomic systems [1]. Myocardial infarction is the irreversible injury of myocardium due to prolonged ischemia caused by coronary arteries narrowing or spasm. Myocardial ischemia/reperfusion (I/R) injury refers to circumstances wherein the ischemic myocardium resumes normal perfusion, but tissue damage is worse. Function preservation of infarcted myocardium remains a challenge for physicians [2]. Although great effort has been devoted to basic and clinical research about I/R injury, pathological mechanisms remain poorly defined [3].

I/R injury is a result of multiple factors, such as oxidative stress, intracellular Ca^2+^ overload, inflammation and metabolic derangements [4], most of which perturb the normal function of the endoplasmic reticulum (ER) and cause ER stress. ER is a subcellular organelle involved in the synthesis, maturation and transport of various proteins. Accumulation of misfolded protein in the ER leads to the unfolded protein response (UPR) to rescue the unbalanced ER protein folding ability and rebuild ER homeostasis [5,6,7,8]. Through three discrete signaling branches, PERK, IRE1/Xbp1s and ATF6, the UPR attenuates protein translation, degrades misfolded proteins and increases the production of molecular chaperones involved in protein folding [9]. However, the UPR may promote apoptosis under prolonged stress.

Lysophosphatidylcholines (LPCs), also called lysolecithins, are a class of lipid biomolecules derived from the cleavage of phosphatidylcholine (PC) by phospholipase A2 (PLA2). LPCs accumulate in the heart during myocardial I/R injury, and some LPC species can be used as diagnostic markers of myocardial infarction [10,11]. Intracellular membrane-associated calcium-independent phospholipase A2 beta (iPLA_2_β) catalyzes the conversion between phospholipid and fatty acids and LPC, and participates in multiple pathophysiological processes, such as apoptosis [12,13,14,15]. This enzyme, originally isolated from myocardial tissue, exhibits several specific characteristics due to its activation during ischemia, including independence of calcium, a preference for plasmalogen phospholipids containing arachidonate at the sn-2 position, and inhibition by calmodulin (CaM) and an interaction with ATP in the presence of Ca^2+^. The cellular localization of iPLA_2_β is tissue-specific and dynamically variable [16,17]. Additionally, iPLA_2_β translocates to different organelles, including the plasma membrane, ER, mitochondria and nuclear membrane, and exerts different roles.

Here, we showed that the expression of iPLA_2_β was upregulated in the heart during myocardial I/R injury, and inhibition of iPLA_2_β reduced ER stress and myocardium damage. iPLA_2_β may therefore represent a promising target to ameliorate cardiac damage from ischemic heart disease.

## 2. Materials and Methods

### 2.1. Chemicals and Reagents

Bromoenol lactone (BEL), thapsigargin (TG) and 4-Phenylbutyric acid(4-PBA) were acquired from Sigma-Aldrich (St. Louis, MO, USA).

### 2.2. Primary Neonatal Rat Ventricular Myocyte (NRVM) Isolation, Culture and Transfection

Isolation and culture of NRVMs were conducted as before [18]. In brief, hearts from 1- or 2-day-old Sprague Dawley rats were harvested (provided by the experimental animal center of Sir Run Run Shaw Hospital, affiliated with Zhejiang University). Cardiac ventricles were digested with 0.114% collagenase II (Biosharp, Shanghai, China) and 0.036% trypsin (Solarbio, Beijing, China) at 37 °C. We first pre-plated cells for 90 min to remove non-cardiomyocytes. We then cultured cardiomyocytes in Dulbecco’s modified Eagle’s medium (DMEM) (Cienry, Huzhou, China) supplemented with 10% fetal bovine serum (FBS) (GeminiBio, Woodland, CA, USA) and 0.1 mmol/L bromodeoxyuridine (BrdU) (Sigma, St. Louis, MO, USA). BrdU was used to reduce the proliferation of non-cardiomyocytes. After 48 h, NRVMs were cultured in DMEM without serum for another 24 h, and then they were subjected to various treatments. The Animal Welfare Ethics Committee of Sir Run Run Shaw Hospital, affiliated with Zhejiang University, approved all animal experiments. For iPLA_2_β knockdown, NRVMs were transfected with control scrambled siRNA or siiPLA_2_β (Sigma, St. Louis, MO, USA) mixed with Lipofectamine RNAiMax (Invitrogen, Waltham, MA, USA), based on the manufacturer’s recommendations.

### 2.3. Simulated Ischemia/Reperfusion (sI/R) and Cell Death Assay

To simulate ischemia/reperfusion in vitro, NRVMs were washed with 1X Phosphate Buffer Saline (PBS) two times and then cultured in freshly prepared ischemic Esumi buffer [19]. Next, cells were placed in a hypoxic chamber (StemCell Technologies, Vancouver, Canada) filled with 94% N_2_/5% CO_2_/1% O_2_ for 15 min at a rate of 20 ml/min. The chamber was sealed and kept at 37 °C for an additional 3 h. Next, we replaced the ischemic Esumi buffer with DMEM. The control group was cultured in normal DMEM. Cell death was assessed by lactate dehydrogenase (LDH) assay using Cytotoxicity LDH Assay kits according to the manufacturer’s recommendations (Dojindo, Kumamoto, Japan).

### 2.4. Generation of iPLA_2_β Knockout (KO) Mice

iPLA_2_β knockout (*Pla2g6*^−/−^) mice were generated by Cyagen Biosciences (Suzhou, China) with global deletion of exons 3 and 4 in the *Pla2g6* gene. iPLA_2_β-KO mice were bred to the C57BL/6 background. Homozygous male iPLA_2_β-KO mice of 12 to 14 weeks old were selected as the experimental group and littermate male wild-type (WT) mice were used as the control group. Genotyping primers for the *Pla2g6* null allele were: forward: 5′-GTCCACACTGGCTTGTTCCTTTAG-3′ and reverse: 5′-TGTGCCGATGTCTCTGCTAGGTAG-3′. All animal experiments were approved by the Animal Welfare Ethics Committee of Sir Run Run Shaw Hospital, affiliated with Zhejiang University.

### 2.5. Mouse Genotyping

The genotypes of the *Pla2g6*^−/−^ mice were identified by PCR. MasterMix (2xTaq Plus) and Super DNA Marker were purchased from CoWin Biosciences (Beijing, China) (cat. No. CW2849 and CW2583). Wild-type primers were as follows: forward primer: 5′-GTCCACACTGGCTTGTTCCTTTAG-3′, reverse primer: 5′-CTCTCCTCTACACTATGACATGATC-3′, with a PCR product size of 573 bp. The mutant primers were as follows: forward primer: 5′-GTCCACACTGGCTTGTTCCTTTAG-3′, reverse primer: 5′-TGTGCCGATGTCTCTGCTAGGTAG-3′, with a PCR product size of 650 bp. PCR conditions were as follows: denaturation at 94 °C for 3 min, 94 °C for 30 s, 60 °C for 35 s and 72 °C for 35 s for 30 cycles, followed by extension at 72 °C for 5 min. Agarose gels (2%) were used for electrophoresis. Images were obtained with a Gel Doc™ XR system (Bio-Rad, Hercules, CA, USA).

### 2.6. Cardiac Ischemia/Reperfusion

Male mice (12–14-week-old) mice were subjected to cardiac ischemia/reperfusion (I/R) surgery as previously reported [20]. In brief, mice were anesthetized with isoflurane and placed on a heating pad to keep their body temperature between 34 °C and 37 °C. After intubation with an 18-G tracheal tube, mice were ventilated using a MiniVent mouse ventilator (tidal volume: 260 μl/stroke; ventilation rate: 100 strokes per min). An oblique incision was made from the left sternal border to observe the fourth intercostal space. Under the exposure of the left anterior descending artery, a 6–0 silk suture was tied at a position 2-mm lower than the tip of the left auricle in a loose double knot, and the thread was kept outside the chest. After 30 min of coronary occlusion, the ligation was untied in order to reestablish flow. Sham groups were subjected to only thoracotomy and closure. After 6, 24, or 72 h, animals were sacrificed for tissue collection or 2,3,5-triphenyltetrazolium chloride (TTC) staining. Infarcted area and area at risk (AAR) were quantified, and relative ratios were calculated. For tissue harvesting, left ventricles were divided into ischemic region, boundary region, and distal region. All tissues were snap-frozen in liquid nitrogen and stored at −80 °C until use.

### 2.7. Echocardiography

A Vevo 2100 system (MS400C probe, VisualSonics, Toronto, ON, Canada) was used to examine cardiac contractile function with conscious, mildly restrained mice. M-mode recordings were collected and analyzed to calculate left ventricular end-diastolic dimension (LVIDd) and left ventricular end-systolic dimension (LVIDs). Ventricular fractional shortening (FS%) was calculated as (LVIDd-LVIDs)/LVIDd. All measurements were performed at the papillary muscle level.

### 2.8. TUNEL Staining

Frozen heart sections and cultured cardiomyocytes were stained with terminal deoxynucleotidyl transferase-mediated dUTP nick-end labeling (TUNEL) using an In Situ Cell Death detection kit (Roche, Basel, Switzerland to detect apoptosis. Cryosections (5-µm thickness) were fixed with 4% paraformaldehyde and permeabilized with 0.1% Triton X-100. Cell nuclei were stained with 4’,6-diamidino-2-phenylindole (DAPI). Three fields were selected randomly for each sample to calculate the percentage of TUNEL-positive cells.

### 2.9. Collection of Human Blood Samples

Blood samples were collected from patients with acute myocardial infarction (AMI) within 24 h after percutaneous coronary intervention (PCI) at Sir Run Run Shaw Hospital. A control group included healthy people without AMI who visited the hospital for physical examination. Clinical diagnosis of AMI has followed WHO diagnostic criteria: 1. Symptoms of ischemic chest pain; 2. Dynamic evolution of ST-segment and T-wave (ST-T) characteristic in the electrocardiogram, or abnormal Q wave; 3. Serum myocardial enzyme spectrum increased and decreased. If two of these are satisfied, a diagnosis is made. The myocardial enzyme spectrum of patients with chest pain was measured immediately after admission, and serum cardiac troponin T (cTnT) level was much higher than 0.5ug/L, Creatine Kinase MB Isoenzyme (CK-MB) was much higher than 25IU/L. The study was conducted according to the guidelines of the Declaration of Helsinki, and approved by the Medical Ethics Committee of Sir Run Run Shaw Hospital, affiliated with Zhejiang University (20200210-113).

### 2.10. ELISA Assay

Blood samples clotted for 2 h at ambient temperature and were centrifuged for 15 min at 12,000 rpm 4  °C. Serum was collected and frozen at −20  °C until use. The culture medium of NRVMs was centrifuged to remove cell debris and then stored at −20  °C until use. ELISA kits for lysophosphatidylcholine (LPC) were purchased from CLOUD-CLONE CORP. (CCC, USA). LPCs in the serum of AMI patients and the culture medium of the NRVMs were then determined. ELISA kits for iPLA_2_β were purchased from GIVE (Shanghai, China) and serum iPLA_2_β concentration was evaluated according to the manufacturer’s recommendations. Concentrations were calculated through standard curves using a specific curve fitting tool CurveExert 1.4. The study of human blood samples was performed according to the principles outlined in the Declaration of Helsinki. The research protocol was approved by the Medical Ethics Committee of Sir Run Run Shaw Hospital affiliated with Zhejiang University.

### 2.11. Immunoblotting

Cells and tissues were lysed in radioimmunoprecipitation assay (RIPA) lysis buffer (Solarbio, Beijing, China). Samples were then cleared by centrifugation (12,000 rpm, 15 min, 4  °C). Equal amount of proteins (10–20 μg) were separated by SDS-PAGE and transferred to polyvinylidene difluoride (PVDF) membranes (Millipore, Billerica, MA, USA). The following primary antibodies were used: iPLA_2_β (Santa Cruz Biotechnology, Dallas, TX, USA), GRP78 (Santa Cruz Biotechnology, Dallas, TX, USA), calnexin (Abclonal, Wuhan, China), p-IRE1α, ATF6 (Proteintech, Rosemont, IL, USA), ATF4 (Proteintech, Rosemont, IL, USA), p-PERK (Abclonal, Wuhan China), p-eIF2α (Abclonal, Wuhan, China), CHOP, cleaved caspase-3, Bax, p-CaMKII, SERCA2 (Santa Cruz Biotechnology, Dallas, TX, USA), β-actin (MultiSciences, Hangzhou, China), PERK (Abclonal, Wuhan, China), eIF2α (Abclonal, Wuhan, China), CaMKII and GAPDH (MultiSciences, Hangzhou, China). After incubation with the corresponding secondary antibodies conjugated with horseradish peroxidase (HRP) (MultiScience, Hangzhou, China), proteins were detected using a BioRad ChemiDoc MP Imaging system with enhanced chemiluminescence. All other antibodies were purchased from Cell Signaling Technology (Beverly, MA, USA).

### 2.12. Immunofluorescence Staining

NRVMs were subjected to sI/R, followed by fixation in 4% paraformaldehyde for 10 min. Cells were then rinsed with cold PBS three times and permeabilized with 0.5% Triton X-100 (15 min at RT). After three washes with PBS, NRVMs were blocked with 2% bovine serum albumin (BSA) in PBS (30 min at 37  °C). iPLA_2_β antibodies (1:200, Santa Cruz, sc-166616, Dallas, TX, USA) mixed with reticulum antibodies (1:200, Abclonal, A1066, Wuhan, China) were used to incubate with NRVMs overnight at 4 °C. After washing with cold PBS three times, cells were stained with Alexa Fluor-594 goat anti-mouse antibodies mixed with fluorescein isothiocyanate (FITC) in 5% BSA (1 h at 4 °C). NRVMs were then counterstained with 4′, 6-diamidino-2-phenylindole (DAPI)-Fluoromount G (Southern Biotech, Birmingham, AL, USA), and images were obtained using a Leica confocal microscope. Colocalization analysis was conducted as previously reported [21].

### 2.13. Flow Cytometry

For quantification of cell death, NRVMs were digested with 0.05% trypsin (without EDTA) after washing with cold PBS. Cells were concentrated and suspended in 1mL PBS after neutralizing with fetal bovine serum. Annexin V-FITC and propidine iodide (PI) staining solution (Yeasen, Shanghai, China) were added, and suspensions were evaluated using flow cytometry.

### 2.14. RNA Isolation and Realtime PCR

Cardiac RNA was isolated using Tissue RNA Purification Plus Kits (ES Science, Shanghai, China). Approximately 10–20 mg of tissues was used for each sample. RNA from NRVMs was extracted with the Quick-RNA MicroPrep kits (ES Science, Shanghai, China). Total RNA (150–250 ng) was subjected to reverse transcription using PrimeScript RT Reagent Kits (Perfect Real Time) (TaKaRa, Tokyo, Japan). cDNA was then diluted 10-fold with ddH_2_O, and quantitative real-time PCR (qRT-PCR) was performed using 2 μL cDNA and Hieff^®^ qPCR SYBR^®^ Green Master Mix (No Rox) (Yeasen, Shanghai, China) on a LightCycler 480 (Roche). The fold change of relative mRNA expression was calculated using the 2^−ΔΔCt^ method, with 18S RNA as an internal control. The primers for mouse iPLA_2_β were forward-CTGGTGCCCCTGTCTTGAAT, reverse-CGAGAACAAGTTGGTGACGC. The primers for mouse GRP78 were forward-CCTCTCTGGTGATCAGGATA, reverse-CGTGGAGAAGATCTGAGACT. The primers for rat iPLA_2_β were forward-GCCCTGGCCATTCTACACA, reverse-CACCTCATCCTTCATACGGA. The primers for rat iPLA_2_γ were forward-CCTGAAGGAAAAGGAGTGG, reverse-CTTGTTCCTCCACCATCAAT. The primers for rat sPLA_2_-V were forward-TCCCATCCAAGAGAACGAGTC, reverse-GTGCCACATCCACGTTTCTC. The primers for rat cPLA_2_α were forward-AGAACACCTGGGAAGTGTGAG, reverse TGGAATAAAGCCCCTCGCTC. The primers for rat Lp-PLA_2_ were forward-CGTATGCTCAACCCACCTCT, reverse-AGCCAACTCCTAGCAAAGGC. The primers for rat 18S were forward-AAACGGCTACCACATCCAAG, reverse-CCTCCAATGGATCCTCGTTA. The primers for mouse iPLA_2_γ were forward-CATGCCGCTGGATGAATGTG, reverse-CCTTAGGACATGCGGGGTTT. The primers for mouse cPLA_2_α were forward-AGCTTAAGGCAGGAGCTAACC, reverse-AGCATATCGCCAAAGGTCCC. The primers for mouse sPLA_2_-V were forward-AGAGTCTGTCCTCCTGTGTTG, reverse-TTGGGTTCTTTGTAGCCTGGT. The primers for mouse Lp-PLA_2_ were forward-AGGCTGTATGCTCAACCCAC, reverse-TTTGATGTTCTGGTCACTGCAC. The primers for mouse 18S were forward-AGGGTTCGATTCCGGAGAGG, reverse-CAACTTTAATATACGCTATTGG.

### 2.15. Cell Surface Protein Biotinylation and Detection

NRVMs were first subjected to sI/R. Then, cells were rinsed twice with cold PBS. EZ-link^®^ Sulfo-NHS-LC-Biotin (Thermo Fisher Scientific, Waltham, MA, USA) at a concentration of 0.5 mg/mL was added. As a control, the same volume of PBS was used. Plates were gently shaken (30 min at 4  °C). Tris-Cl, pH 7.5 at 100 nM was used to stop biotinylation reaction. After rinsing twice with cold PBS, the cells were lysed with radioimmune precipitation buffer (RIPA). To purify surface proteins, neutravidin agarose beads were mixed with the lysate (overnight at 4  °C). The beads were then washed six times with PBS. Cell surface proteins were released by mixing with SDS-PAGE loading buffer and heating (100  °C for 5 min). Immunoblotting was then conducted to detect iPLA_2_β biotinylation. ATP1A (ATPase Na^+^/K^+^ transporting subunit alpha 1) was used as a cell membrane protein marker and loading control.

### 2.16. Assay for [Ca^2+^]i Levels

For the analysis of [Ca^2+^]i levels, cells were loaded with 5 μmol/L Fluo-4AM (Yeasen, China) at 37 °C for 30 min. The cells were digested and collected, [Ca^2+^]i levels were detected by flow cytometry.

### 2.17. Statistics

Data were expressed as mean ± SEM and Student’s t-test was used to calculate statistical difference between two groups. Differences among multiple groups were analyzed by one-way ANOVA, followed by Tukey’s test. *p* < 0.05 was considered statistically significant.

## 3. Results

### 3.1. Myocardial Ischemia/Reperfusion (I/R) Injury Induces iPLA_2_β In Vivo

**Figure 1:**
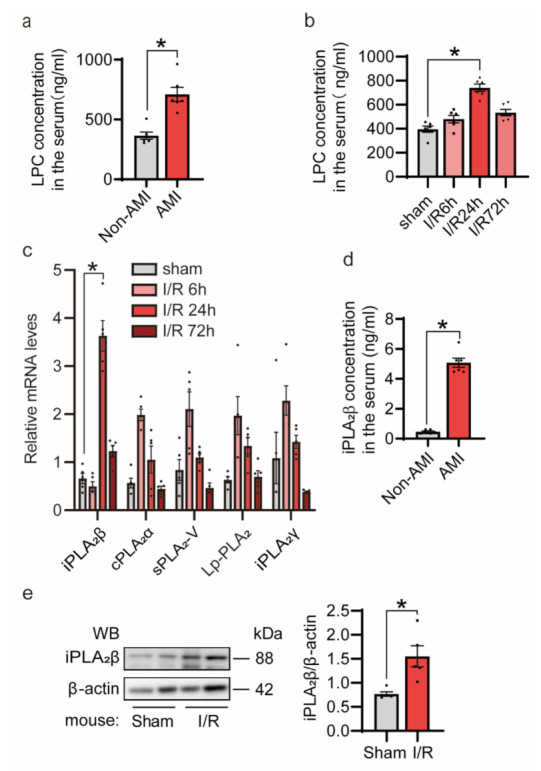
The elevated expression of iPLA_2_β during myocardial ischemia/reperfusion (I/R). (**a**) Lysophosphatidylcholine (LPC) was increased in the sera of the acute myocardial infarction (AMI) patients. Twelve serum samples were collected, six of which were in the AMI group. LPC ELISA was performed to detect the LPC concentration in serum. (**b**) Upregulation of LPC in mouse sera after I/R injury. Mice at 12–14 weeks of age were subjected to myocardial ischemia for 30 min, followed by reperfusion for 6, 24, 72 h. Serum samples were collected, and LPC ELISA was performed to detect the LPC concentration. *n* = 6 per group. (**c**) The mRNA level of phospholipase A2 in mouse hearts upon myocardial I/R was detected. Quantitative RT PCR (qRT-PCR) was conducted to measure mRNA level changes, with 18S as an internal control. *n* = 5 per group. (**d**) iPLA_2_β was upregulated in the sera of the AMI patients. iPLA_2_β ELISA was performed to detect the iPLA_2_β concentration. (**e**) The expression of iPLA_2_β in mouse hearts was increased after I/R. Mice were subjected to myocardial ischemia for 30 min, followed by 24 h of reperfusion. Immunoblot analysis was performed for iPLA_2_β. β-actin was used as a loading control. *n* = 5 per group. Data are represented as mean ± SEM. All experiments were independently replicated in triplicate. * *p* < 0.05.

Previous studies have shown that myocardial I/R injury increases the production of lysophosphatidylcholine (LPC) [22,23,24]. To determine whether LPC is stimulated by myocardial I/R injury in humans, we collected serum samples from 12 patients for ELISA assay. The basic demographic and clinical characteristics of the AMI patients and healthy donors are shown in Table 1. ELISA assay for LPC showed that the serum LPC level was higher in the AMI patients than the non-AMI controls (Figure 1a). 

Next, blood samples from 12–14-week-old mice subjected to myocardial ischemia for 30 min, followed by reperfusion for 6, 24, 72 h were collected for ELISA assay. Our results showed that LPC level was increased in mice after 24 h of reperfusion compared to the sham group (Figure 1b). LPC is generated by partial hydrolysis of phosphatidylcholines, which removes one of the fatty acid groups. This hydrolysis is generally the result of the enzymatic action of phospholipase A2 (PLA_2_). Previous studies have shown that the subtypes of PLA_2_, including cPLA_2_α, sPLA_2_-V, Lp-PLA_2_, iPLA_2_β and iPLA_2_γ, are associated with myocardial I/R injury [25,26,27,28,29,30]. Here, we first examined the correlation between PLA_2_ enzymes and I/R. We found that the mRNA level of iPLA_2_β was elevated significantly after 24 h of reperfusion, which was also consistent with the increase of LPC concentrations (Figure 1c). In contrast, the increase of other PLA_2_ subtypes peaked at 6 h instead of 24 h after I/R. Next, we asked whether iPLA_2_β was also increased at the protein level upon I/R. Indeed, our iPLA_2_β ELISA data showed that serum levels of iPLA_2_β in the AMI patients were significantly higher than those in the non-AMI patients (Figure 1d). Consistently, the protein level of iPLA_2_β in mouse hearts underwent I/R was also dramatically increased (Figure 1e). Taken together, these data indicate that myocardial I/R injury induces iPLA_2_β expression in the heart.

### 3.2. sI/R Induced iPLA_2_β In Vitro

**Figure 2:**
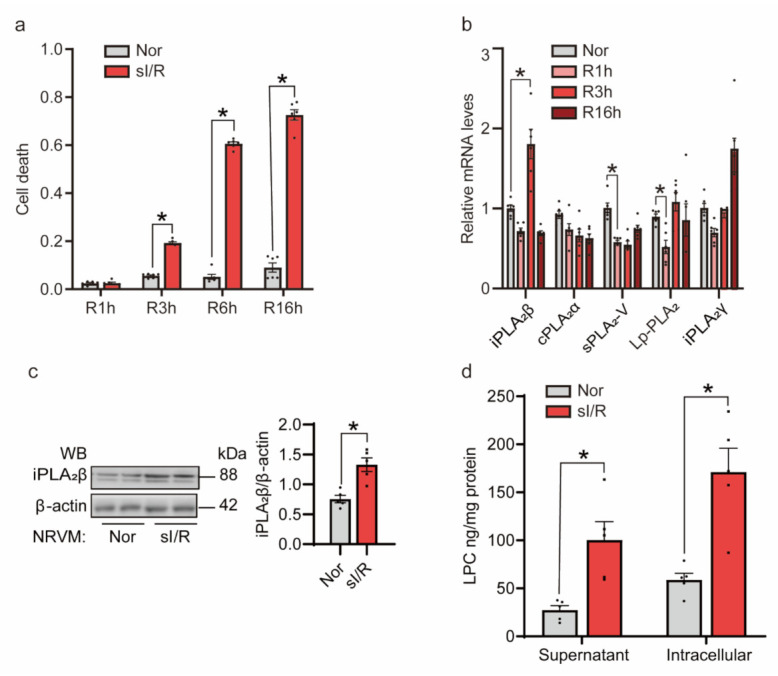
Simulated ischemia/reperfusion (sI/R) induces iPLA_2_β upregulation in neonatal rat ventricular myocytes (NRVMs). (**a**) sI/R caused significant cell death. NRVMs were subjected to 3 h of ischemia followed by 3, 6, 16 h of reperfusion. Lactate dehydrogenase (LDH) was determined in culture medium and in cell lysates, respectively. Relative cell death was calculated based on the ratio of released LDH in the medium. *n* = 6 per group. (**b**) sI/R led to strong induction of phospholipase A2 at the mRNA level. NRVMs were subjected to ischemia for 3 h, followed by different times of reperfusion. qRT-PCR was conducted to measure mRNA changes with 18S as an internal control. *n* = 6 per group. (**c**) sI/R induced the expression of iPLA_2_β at the protein level. Immunoblotting showed that iPLA_2_β was significantly increased at the protein level (left). β-actin was used as a loading control. Quantification is shown on the right. *n* = 5 per group. (**d**) Lysophosphatidylcholine (LPC) was increased during sI/R. NRVMs were subjected to 3 h of ischemia and 3 h of reperfusion. LPC ELISAs were performed to detect LPC concentrations in culture medium and in cell lysates. Relative LPC concentration in cell lysates was normalized based on total cellular protein concentrations. *n* = 5 per group. Data are represented as mean ± SEM. All experiments were independently replicated in triplicate. * *p* < 0.05.

We treated NRVMs using the Esumi ischemic buffer, and incubated the cells in a 94% N_2_/5% CO_2_/1% O_2_ hypoxic chamber for 3 h. Then, the buffer was replaced with a normal medium. We next determined cardiomyocyte death by assessing LDH release. We found that sI/R induced a time-dependent increase of cardiomyocyte death (Figure 2a). We found that, consistent with the elevated LDH release, NRVMs subjected to simulated 3-h ischemia/3-h reperfusion (sI/R) showed a significant increase of iPLA_2_β expression at both mRNA and protein levels (Figure 2b,c). In contrast, the mRNA levels of other types of PLA_2_, including cPLA_2_α, sPLA_2_-V, Lp-PLA_2_ and iPLA_2_γ, had no significant changes (Figure 2b). Besides, LPC concentrations were also upregulated in both supernatants and intracellular extracts (Figure 2d).Following sI/R, we observed that GRP78, an ER stress marker, was increased at both mRNA and protein levels (Supplementary Figure S1a,b). Moreover, NRVMs subjected to sI/R showed an increase of the spliced form of XBP1 mRNA (Supplementary Figure S1c). In summary, these findings suggest that sI/R leads to upregulation of iPLA_2_β and induction of the UPR.

### 3.3. Knockout of iPLA_2_β Alleviated Heart I/R Injury

**Figure 3:**
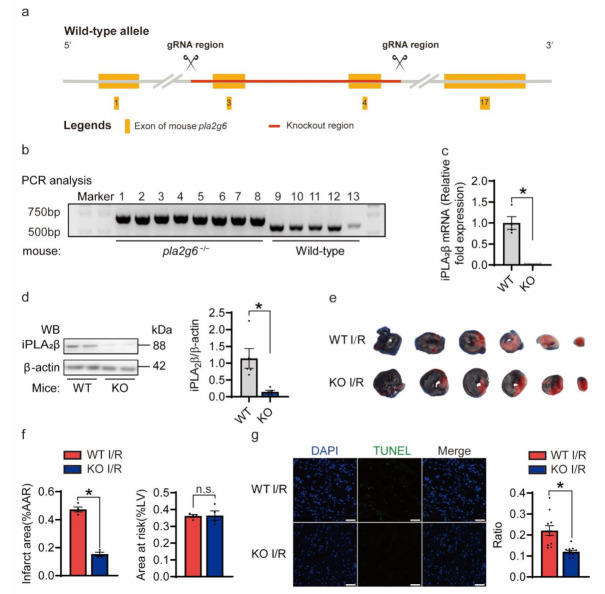
Knockout of iPLA_2_β protects the heart from I/R injury. (**a**) Overview of the targeting strategy to create a *Pla2g6*^−/−^ mouse model (C57BL/6N) by CRISPR/Cas9-mediated genome engineering. (**b**) Knockout (KO) mice were genotyped by agarose gel electrophoresis. The size of the PCR product of *Pla2g6*^−/−^ mice was 650 bp, whereas the PCR product of the wild-type (WT, *Pla2g6*^+/+^) mice was 573 bp. (**c**) Absence of iPLA_2_β mRNA in KO mice. Total RNA was isolated from ventricular tissues of WT and iPLA_2_β KO mice, respectively. Real-time PCR was conducted to assess iPLA_2_β at the mRNA level. *n* = 4–5 for each group. (**d**) Confirmation of iPLA_2_β deletion in iPLA_2_β-KO mice. Total proteins were extracted from the ventricular tissues of wild-type (WT) and iPLA_2_β-KO mice. Western blotting was conducted to assess iPLA_2_β at protein level. *n* = 5 for each group. (**e**,**f**) Knockout of iPLA_2_β protected the heart from I/R injury. WT and iPLA_2_β-KO mice at 12–14 weeks of age were subjected to myocardial ischemia for 30 min, followed by 24 h of reperfusion. Then, 2,3,5-triphenyltetrazolium chloride (TTC) staining was conducted to detect and quantify the injured cardiac tissue. The white region depicts the ischemic infarct zone; the pink/red area is the border zone; and the blue region represents the remote zone. The relative ratio of the infarct region (IF) to the area at risk (AAR, ischemic and border) was compared between WT and iPLA_2_β-KO mice. The relative ratio of AAR to the left ventricle did not differ between WT and KO groups, suggesting that I/R surgery was performed similarly across genotypes. *n* = 4 per group. (**g**) Representative photographs of heart sections stained with terminal-deoxynucleotidyl transferase-mediated dUTP nick end labeling (TUNEL) from WT and KO mice after myocardial I/R (left). Scale bar = 25 μm. Quantification is shown on the right. *n* = 10–11 per group. Data are represented as mean ± SEM. All experiments were independently replicated in triplicate. * *p* < 0.05.

To explore the role of iPLA_2_β in vivo, we generated a *Pla2g6* (the gene encoding iPLA_2_β) knockout (KO) mouse model (C57BL/6N) by CRISPR/Cas9-mediated genome engineering (Figure 3a). Positive mice were identified by PCR screening, and the size of the PCR products of *Pla2g6^−/−^* mice was 650 bp, whereas PCR products for the wild-type (WT, *Pla2g6^+/+^*) mice were 573 bp. Mice designated #1–8 were homozygous *Pla2g6^−/−^* mice (Figure 3b), and mice designated #9–13 were homozygous *Pla2g6^+/+^* mice. To confirm the knockout of *Pla2g6*, we performed qRT-PCR and Western blotting. iPLA_2_β-KO mice showed significantly lower iPLA_2_β expression at both mRNA level and protein levels (Figure 3c,d). Furthermore, WT and *Pla2g6^−/−^* mice had similar body weights (Supplementary Figure S2a), heart rates (Supplementary Figure S2b), ventricular sizes and contractile functions (Supplementary Figure S2c) at baseline. Next, 30-min of myocardial ischemia and 24-h of reperfusion were performed with 12–14-week-old WT control mice and KO mice. We used TTC staining to identify and quantify damaged tissue areas (Figure 3e). The relative ratios of infarct area to area at risk (AAR) of the WT and KO hearts were calculated and compared. iPLA_2_β deletion led to a significantly smaller infarct size (Figure 3f). In addition, there was no significant difference in the relative ratio of AAR to the left ventricle between control group and KO group, suggesting that I/R surgery was similarly conducted between groups. Moreover, echocardiographic analysis showed iPLA_2_β deficiency resulted in improved systolic and diastolic function 24 h after I/R surgery (Table 2), which indicates that knockout of iPLA_2_β preserves cardiac performance in respond to I/R injury. Furthermore, heart sections stained with TUNEL showed less myocardial apoptosis in iPLA_2_β KO mice than control littermates (Figure 3g). Taken together, our data indicate that knockout of iPLA_2_β protects the heart against I/R injury.

### 3.4. Inhibition of iPLA_2_β Ameliorates NRVMs Death Triggered by sI/R In Vitro

**Figure 4:**
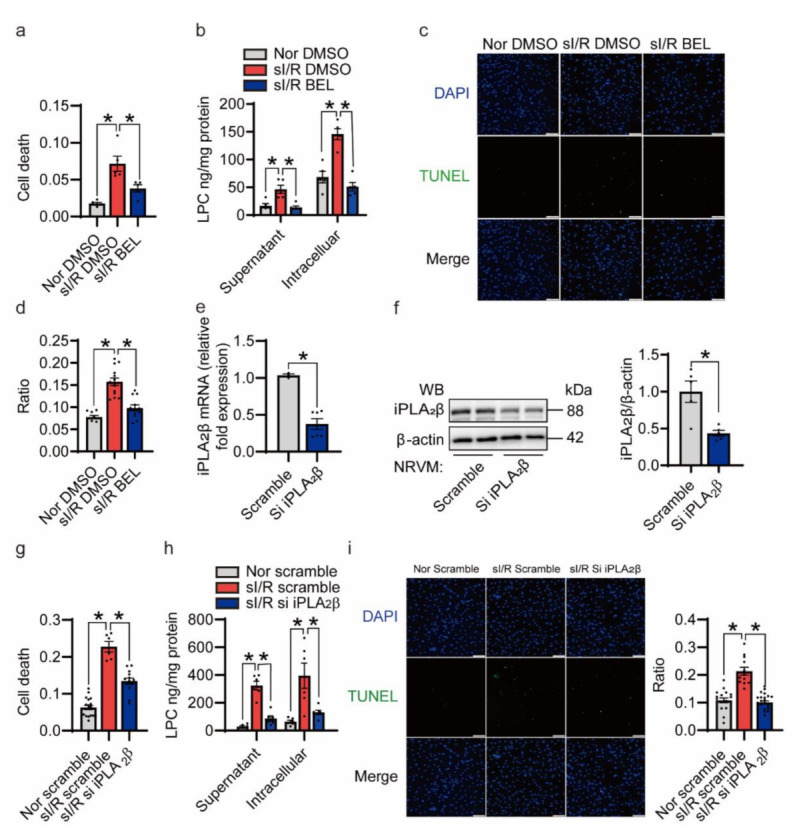
Inhibition of iPLA_2_β by bromoenol lactone (BEL) or knockdown of iPLA_2_β protects NRVMs from sI/R-induced cell death. (**a**) Inhibition of iPLA_2_β blunts sI/R-induced cell death. NRVMs were subjected to ischemia for 3 h and then pretreated with 15 µM BEL for 15 min followed by 3 h of reperfusion. LDH levels were measured to determine relative cell death. *n* = 5 per group. (**b**) Inhibition of iPLA_2_β decreased sI/R-induced LPC release. After 3 h of ischemia, NRVMs were treated with BEL, followed by reperfusion for 3 h. Relative LPC concentrations were determined with LPC ELISA kits. *n* = 5 per group. (**c**) Inhibition of iPLA_2_β extenuated sI/R-induced cell apoptosis as determined by terminal-deoxynucleotidyl transferase-mediated dUTP nick end labeling (TUNEL)-positive cardiac myocytes. Apoptotic nuclei were identified by TUNEL staining (green), and total nuclei were identified by 4’,6-diamidino-2-phenylindole (DAPI) counterstaining (blue). Representative photomicrographs are shown. Scale bar = 20 μm. (**d**) Quantitative analysis of TUNEL-positive cells under the normoxic or sI/R condition. *n* = 6–14 per group. (**e**) The relative gene expression of iPLA_2_β decreased in the siiPLA_2_β-transfected NRVMs. qRT-PCR was conducted to examine mRNA level changes with 18S as an internal control. *n* = 3–6 per group. (**f**) Immunoblot analysis for iPLA_2_β. β-actin was used as a loading control. *n* = 5 per group. (**g**) Knockdown of iPLA_2_β attenuated sI/R-induced cell death. LDH levels were measured to determine relative cell death. *n* = 6–16 per group. (**h**) Knockdown of iPLA_2_β decreased sI/R-induced LPC release. After transfection with siiPLA_2_β, NRVMs were subjected to 3 h of ischemia and 3 h of reperfusion. Relative LPC concentrations were examined with LPC ELISA kits. *n* = 6 per group. (**i**) Knockdown of iPLA_2_β attenuated sI/R-induced cell apoptosis as determined by TUNEL-positive cardiac myocytes. Representative photomicrographs are shown on the left. Scale bar = 20 μm. Quantification is shown on the right. *n* = 11–18 per group. Data are represented as mean ± SEM. All experiments were independently replicated in triplicate. * *p* < 0.05.

To investigate the role of iPLA_2_β in I/R in vitro, we adopted an inhibitor-based approach. First, after 3 h of ischemia, we inhibited iPLA_2_β in NRVMs with 15 µM bromoenol lactone (BEL) for 15 min, which is a potent and irreversible inhibitor of iPLA_2_β [31]. DMSO was used as a negative control. Our results showed that inhibition of iPLA_2_β significantly reduced cell death caused by sI/R (Figure 4a). LPC ELISAs verified that BEL pretreatment reduced sI/R induced supernatant and intracellular LPC release, which was mainly due to the reduced activity of iPLA_2_β (Figure 4b). Additionally, the decreased number of TUNEL-positive cells in the BEL-treated group confirmed that the inhibition of iPLA_2_β protected NRVMs from the sI/R-induced cell death (Figure 4c,d). Recent studies have shown that BEL inhibits other key enzymes in phospholipid metabolism as well [31]. To further test the function of iPLA_2_β in sI/R injury directly, we transfected NRVMs with siiPLA_2_β to knock down iPLA_2_β. SiiPLA_2_β efficiently decreased target gene expression at both protein and mRNA levels (Figure 4e,f). Knockdown of iPLA_2_β modestly but significantly decreased cell death, as shown by an LDH release assay and the LPC concentration measurement (Figure 4g,h). Furthermore, knockdown of iPLA_2_β mitigated NRVM apoptosis caused by sI/R as shown by TUNEL assays (Figure 4i). Collectively, these results demonstrate that inhibition of iPLA_2_β protects cardiomyocytes from sI/R injury in vitro.

### 3.5. The Reduction of I/R Injury Is Mainly Achieved by Reducing Apoptosis

**Figure 5:**
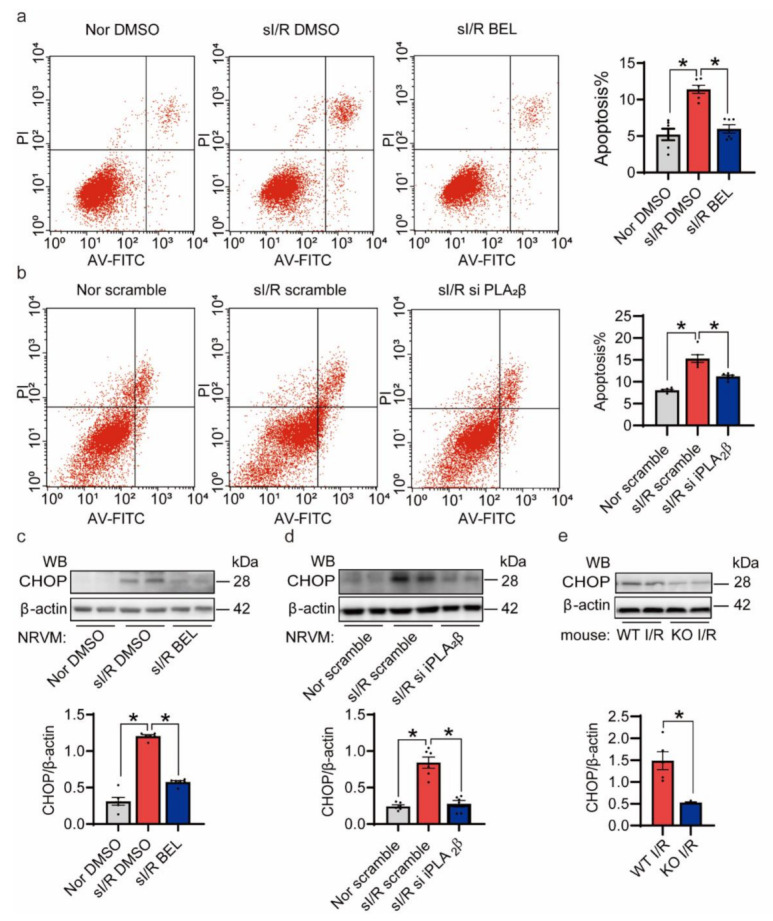
Inhibition of iPLA_2_β alleviates I/R injury by reducing apoptosis. (**a**) Suppression of iPLA_2_β by BEL conferred significant protection against I/R-induced cell death as determined by flow cytometry of Annexin V- and PI-positive cells. A representative histogram is shown. Quantification is shown on the right. *n* = 6 per group. (**b**) Knockdown of iPLA_2_β protected NRVMs against I/R-induced cell death as determined by flow cytometry of Annexin V- and PI-positive cells. A representative histogram is shown. Quantification is shown on the right. *n* = 6 per group. (**c**) The expression of CHOP was detected and quantified by Western blotting. *n* = 6 per group. (**d**) The expression of CHOP was reduced by transfection with siiPLA_2_β. *n* = 6 per group. (**e**) CHOP expression in the iPLA_2_β-KO mouse heart was reduced upon I/R injury. β-actin was used as a loading control. *n* = 5 per group. Data are represented as mean ± SEM. All experiments were independently replicated in triplicate. * *p* < 0.05.

Previous work suggests that apoptosis is involved in myocardial I/R injury [32]. Since prolonged ER stress leads to apoptosis, we hypothesized that the inhibition of iPLA_2_β might alleviate I/R injury caused-myocardial damage by reducing apoptosis. Indeed, flow cytometric analysis showed that the inhibition of iPLA_2_β protected NRVMs from sI/R injury, mainly by decreasing Annexin V+/PI+-positive apoptotic cells (Figure 5a,b). We observed that the expression of CHOP was markedly reduced in the BEL-pretreated and siiPLA_2_β-transfected NRVMs under the sI/R condition, respectively (Figure 5c,d). Moreover, the expression of CHOP in iPLA_2_β-KO mouse hearts after I/R injury was downregulated compared to that in WT mouse hearts (Figure 5e). Additionally, there was no significant difference of CHOP expression at baseline between WT and iPLA_2_β-KO mice (Supplementary Figure S3a).

### 3.6. iPLA_2_β Translocation to the ER Aggravates ER Stress-Induced Apoptosis during I/R

**Figure 6:**
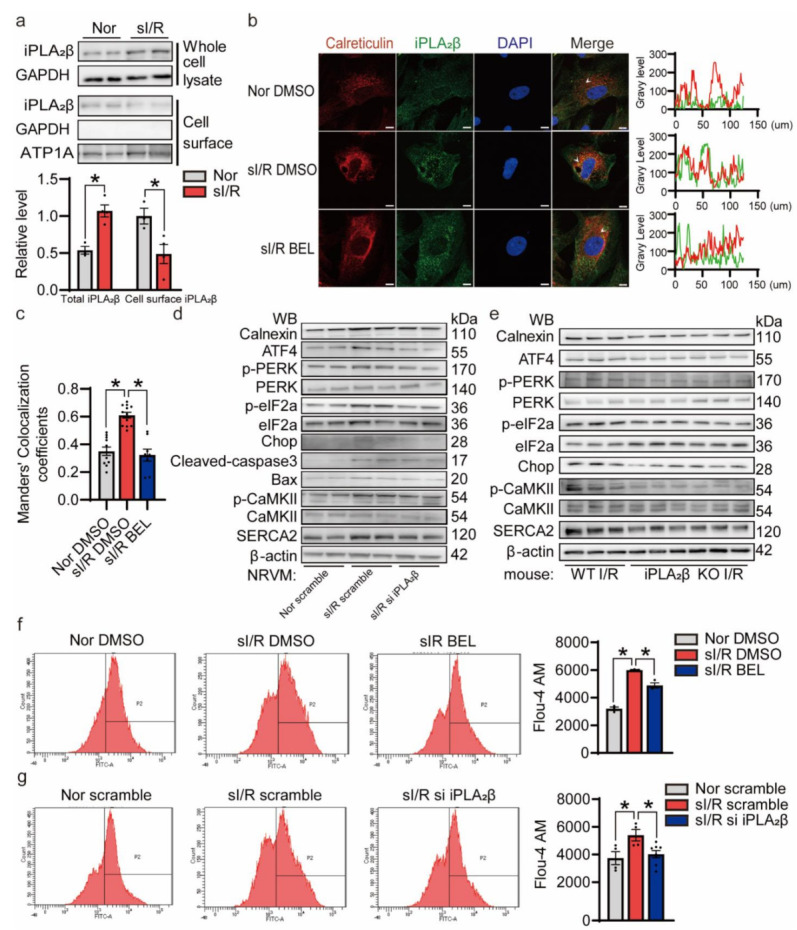
I/R promotes iPLA_2_β translocate to the ER, leading to cardiomyocyte apoptosis induced by ER stress. (**a**) sI/R caused no significant increase in iPLA_2_β on the cell surface membrane. NRVMs were subjected to sI/R and then placed on ice. EZ-link Sulfo-NHS-LC-Biotin was used to modify cell surface-localized proteins. After isolation of biotinylated surface proteins with neutravidin, immunoblotting was conducted to detect iPLA_2_β. ATP1A was used as loading control of cell-surface-localized proteins. Quantification (below) revealed no increase in cardiomyocyte surface-localized iPLA_2_β after sI/R. *n* = 3–4 for each group. (**b**) iPLA_2_β was localized in the ER. iPLA_2_β was detected by confocal immunofluorescence staining (green). ER was revealed by confocal immunofluorescence staining for calreticulin (red). The areas of interest are labeled with white arrows. The signaling intensity for both channels was scanned and recorded. Results are shown on the right. Scale bar = 20 μm. (**c**) Colocalization of iPLA_2_β and ER was quantified by Mander’s colocalization coefficients (MCCs). The comparison suggests that the inhibition of iPLA_2_β significantly disrupts iPLA_2_β and ER colocalization. *n* = 8–12 per group. (**d**) Knockdown of iPLA_2_β relieved ER stress caused by I/R. Immunoblotting analysis was performed to detect the ER stress markers and Ca^2+^ -regulated related proteins. β-actin was used as a loading control. The protein levels of calnexin, ATF4, p-PERK, p-eIF2a, CHOP, cleaved-caspase3, Bax, p-CaMKII and SERCA2 were downregulated in the siiPLA_2_β -transfected NRVMs. (**e**) Knockout of iPLA_2_β relieved the ER stress caused by I/R. Immunoblotting analysis of ER stress markers and Ca^2+^-regulated related proteins were performed. β-actin was used as a loading control. The protein levels of calnexin, ATF4, p-PERK, p-eIF2a, CHOP, p-CaMKII and SERCA2 were downregulated in the *Pla2g6* KO mouse heart tissues. (**f**) Inhibition of iPLA_2_β by BEL reduced calcium overload upon sI/R. Intracellular Ca^2+^ was measured using 5 μM Fluo-4 AM. Mean fluorescence intensity indicates the concentration of intracellular Ca^2+^. Quantification is shown on the right. *n* = 3 per group. (**g**) Knockdown of iPLA_2_β reduced calcium overload during sI/R. Intracellular Ca^2+^ was measured using 5μM Fluo-4 AM. Mean fluorescence intensity indicates the concentration of intracellular Ca^2+^. Quantification is shown on the right. *n* = 4–7 per group. Data are represented as the mean ± SEM. All experiments were independently replicated in triplicate. * *p* < 0.05.

The function of iPLA_2_β is influenced by its organelle localization, which is regulated by the intracellular environment [16]. To determine the localization of iPLA_2_β in cardiomyocyte upon I/R injury, a biotinylation experiment for cell surface proteins was conducted. sI/R did not increase the cell membrane localization of iPLA_2_β, which actually showed a trend of decrease (Figure 6a). Immunofluorescence was next used to investigate whether iPLA_2_β was present on the ER membrane. We found that colocalization of iPLA_2_β and an ER marker calreticulin was significantly increased during sI/R. In contrast, iPLA_2_β inhibition significantly disrupted iPLA_2_β and ER colocalization (Figure 6b,c). The upregulation of ER stress markers, apoptotic markers and Ca^2+^ regulated related proteins induced by sI/R was decreased by the inhibition of iPLA_2_β in vitro and in vivo (Figure 6d,e). Quantification of these blots were shown (Supplementary Figure S4a,b). However, significant differences were not observed in the other two UPR marker proteins, ATF6 and IRE1α (Supplementary Figure S5a,b). When NRVMs were subjected to 0.5 µM thapsigargin (TG), an ER stress activator, for 6 h, we also found that colocalization of iPLA_2_β with ER was significantly increased under ER stress condition (Supplementary Figure S5d). Inhibition of iPLA_2_β reduced intracellular Ca^2+^ during sI/R (Figure 6f,g). Moreover, inhibition of ER stress by 5 mM 4-phenylbutyric acid (4-PBA) for 30 min ameliorated cardiomyocyte death caused by sI/R (Supplementary Figure S5c). Collectively, these findings suggest that I/R induces iPLA_2_β expression and promotes its translocation to the ER, which leads to unbalanced lipid metabolism homeostasis, aggravates calcium overload, triggers ER stress and eventually causes cardiomyocyte apoptosis. 

## 4. Discussion

Coronary heart disease is caused by atherosclerotic lesions in the coronary arteries that narrow or block the lumen of the vessels, resulting in myocardial ischemia, hypoxia, or necrosis. Reports from the World Health Organization’s website in 2017 show that cardiovascular disease (CVD) is the primary cause of death globally: more people die annually from CVD than from any other causes. Of these deaths, 85% are due to heart attack and stroke, with ischemic heart disease as the predominating one. The current development of PCI technology makes it possible to reduce the myocardial infarction area effectively and improve clinical outcomes. However, the mortality rate of patients with myocardial infarction still remains high. Studies have shown that even the restoration of blood flow to the ischemic myocardium cannot significantly reduce the infarct area, instead, further damage has been observed. This phenomenon is referred to as cardiac ischemia/reperfusion (I/R) injury. The pathological mechanism of cardiac I/R injury includes the accumulation of oxygen free radicals [33], calcium iron overload [34], energy metabolism failure [35], cell apoptosis [36] and other pathological factors. Cardiac I/R injury is a complex and multi-factorial pathological process that needs further investigations.

**Figure 7:**
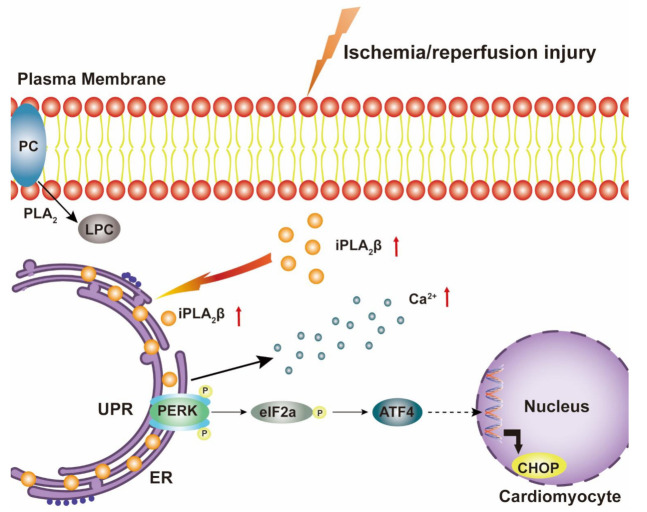
Illustration of the role of iPLA_2_β on ER stress in cardiomyocyte upon I//R. PLA_2_ expression is up-regulated in cardiomyocyte after I/R, which consequently leads to an increase of phosphatidylcholine (PC) metabolism. The generation of LPC is elevated and cardiomyocyte damage is augmented. The protein level of intracellular iPLA_2_β, one of the PLA_2_ enzymes, is upregulated. The increased iPLA_2_β translocates to the ER and induces ER stress, which further leads to apoptosis through the PERK/ATF4/CHOP pathway. Additionally, iPLA_2_β may also increase intracellular calcium flow in cardiomyocyte.

ER stress has been proven to be critical for the progression of cardiac diseases, including I/R [37,38,39]. In our study, we revealed the important effects of iPLA_2_β on ER stress during myocardial I/R injury. The levels of iPLA_2_β and ER stress markers were induced by sI/R injury, with a concomitant increase of apoptotic markers. Besides, inhibition of iPLA_2_β in cardiomyocytes decreased cardiomyocyte death in response to sI/R injury via the ATF4/CHOP pathway. These results indicate that iPLA_2_β increased upon I/R injury caused ER stress and further induced apoptosis. Moreover, iPLA_2_β-deficient mice showed alleviated myocardial I/R injury. We also found that iPLA_2_β participated in ER stress-induced apoptosis by translocating to the ER (Figure 7). Based on these, we propose that iPLA_2_β is critical for the control of ER stress and cardiac function by its dynamic cellular localization.

iPLA_2_β, which is also referred to as PLA_2_ group VI and encoded by *PLA2G6*, contributes to the regulation of the concentration of phosphatidylcholine, a metabolite abundant in the cell membrane. iPLA_2_β is also involved in multiple processes of phospholipid remodeling, arachidonic acid release [40], Fas receptor-mediated apoptosis [41,42], and transmembrane ion flux [43]. Mutations in *PLA2G6* have been proven to lead to mitochondrial dysfunction [44] and related neurological diseases [45,46,47,48], including Parkinson’s disease 14 [49] and hereditary spastic paraplegia [50]. Intracellular localization of iPLA_2_β is dynamic and tissue-specific [51,52]. Previous studies have shown that proinflammatory cytokines induce iPLA_2_β upregulation-caused cell apoptosis by stimulation of ER stress in β-cells, an effect blocked by iPLA_2_β inhibitor bromoenol lactone (BEL) [53].

iPLA_2_β and cardiovascular function are closely related. iPLA_2_β activation in the heart may result in both positive and negative effects [14,54,55]. The inhibition of iPLA_2_ signaling pathway blocks the negative inotropic effect of HIV gp120 on cardiac myocytes [56], and activation of iPLA_2_β has been reported to result in reduced conduction of arrhythmias caused by diabetic heart ischemia [57]. iPLA_2_β also regulates calcium flow in various cells [58,59,60,61]. For example, iPLA_2_ and its metabolite LPC play a key role in the control of endothelial store-operated Ca^2+^ entry and arterial tone [62].

It has been reported that the UPR is closely associated with lipid metabolism imbalance in the ER [63]. The UPR has been shown to be activated by changes in different lipids, which is independent of protein folding homeostasis in the ER lumen [64]. In this study, we established the heart I/R injury model in vivo and in vitro and observed that the activation of ER stress was accompanied by the upregulation of iPLA_2_β. In view of the metabolic function of membrane phospholipids, we hypothesized that changes of iPLA_2_β in the ER would lead to the disruption of ER function and thus cause ER stress. We confirmed that the increase of iPLA_2_β in the ER lumen induced ER stress, caused calcium overload, and ultimately led to apoptosis. However, we did not determine the mechanism of iPLA_2_β’s translocation into ER under I/R, nor did we identify the ER protein that acts directly with it. Further studies are required to answer these key questions. iPLA_2_β is intensively studied in the field of the nervous system [65], and it has been reported that iPLA_2_β’s pathogenic causes are related to the structural and functional changes of the ER [66] and mitochondria [67]. Scoot et al. showed that iPLA_2_β localizes in the mitochondria and affects the phospholipid metabolism of the mitochondrial membrane during myocardial I/R injury, which leads to the impairment of mitochondrial structure and function and consequently causes cardiomyocyte death [68]. Although many studies have confirmed the relationship between iPLA_2_β and ER stress, our study contributes to the understanding of the connection between iPLA_2_β and myocardial I/R injury. In addition, studies have reported that iPLA_2_β is essential for maintaining basal ER homeostasis, suggesting that this enzyme performs different functions under pathological and physiological conditions.

In summary, our study identified a key role of iPLA_2_β in ER stress-induced apoptosis and in intracellular calcium homeostasis upon cardiac I/R. Inhibition of iPLA_2_β reduces myocardial I/R injury and preserves cardiac function. Therefore, inactivation of iPLA_2_β may serve as a potential therapeutic strategy for myocardial ischemia/reperfusion injury.

## 5. Limitation

We detected LPC concentration in the sera of patients with acute myocardial infarction by ELISA. Although previous reports have shown no significant difference in the detection results between ELISA and HPLC methods [69,70], it is undeniable that HPLC is more accurate, stable and specific than ELISA [71]. LPCs are a class of metabolites derived from phosphatidylcholines, which are composed of a variety of species [72]. Due to the unavailability of HPLC, we could not detect and analyze the various LPC components.

The neonatal cardiomyocytes were chosen for in vitro experiments in our study. There are some limitations in using neonatal cardiomyocytes as a model system. First, the phenotypic immaturity of neonatal cardiomyocytes limits the full translation of the findings to the in vivo conditions, for example, metabolic characteristics of neonatal cells are different from those in adult cardiomyocytes [73]. Secondly, the lack of extracellular matrix in the culture system may alter the behavior of these cells, resulting in a certain discrepancy between in vitro and in vivo findings [74].

## Figures and Tables

**Table 1:** Basic demographic and clinical characteristics of patients in two groups (*n* = 12).

	Non- AMI Group (*n* = 6)	AMI Group (*n* = 6)
Age (years)	54.83 ± 4.79	55.00 ± 8.72
Gender (female: male)	3:3	2:4
BMI	21.32 ± 2.32	24.77 ± 5.73
Hypertension (Yes: No)	2:4	3:3
Diabetes (Yes: No)	3:3	1:5
Smoke (Yes: No)	2:4	4:2
SBP (mmHg)	119.00 ± 11.05	121.83 ± 17.27
DBP (mmHg)	67.50 ± 10.31	77.50 ± 17.41
TG (mmol/L)	1.59 ± 1.16	1.40 ± 0.57
CHO (mmol/L)	5.13 ± 1.20	4.03 ± 1.11
HDL (mmol/L)	1.02 ± 0.15	1.04 ± 0.14
LDL (mmol/L)	2.25 ± 1.15	1.87 ± 0.62

Data are expressed as mean ± SD; AMI: acute myocardial infarction; BMI: body mass index; SBP: systolic blood pressure; DBP: diastolic blood pressure; TG: triglyceride; CHO: cholesterol; HDL: high density lipoprotein; LDL: low density lipoprotein Hypertensive patients usually take antihypertensive drugs to control their blood pressure.

**Table 2:** Echocardiographic measurements for iPLA_2_β-KO and WT mice at 24 hours after I/R operation.

Parameter	WT I/R (*n* = 5)	KO I/R (*n* = 5)
IVS (mm)	1.00 ± 0.17	0.85 ± 0.96
LVPWs (mm)	1.08 ± 0.14	1.07 ± 0.22
LVIDd (mm)	3.65 ± 0.26	3.05 ± 0.41 *
LVIDs (mm)	3.22 ± 0.26	2.40 ± 0.37 *
FS (%)	11.79 ± 2.69	21.46 ± 2.26 *
EF (%)	26.02 ± 5.54	44.91 ± 4.26 *

IVS, interventricular septum thickness; LVPW, left ventricular posterior wall thickness; LVIDd, left ventricular end-diastolic dimension; LVIDs, left ventricular end- systolic dimension; FS, fractional shortening; EF, ejection fraction. * *p* < 0.05 versus WT I/R group.

## Data Availability

Data presented in the article are available by request to first author, Tingting Jin (11818361@zju.edu.cn).

## Supplementary Materials

The following materials are available online at https://www.mdpi.com/article/10.3390/cells10061446/s1, Supplement Figure S1. Simulated ischemia/reperfusion (sI/R) injury-triggered ER stress in NRVMs. Supplement Figure S2. Analysis of *Pla2g6*^−/−^ mice and wild-type mice. Supplement Figure S3. No significant difference in the expression of CHOP at the basal level between WT and KO mice. Supplement Figure S4. The inhibition of iPLA_2_β in vitro and in vivo decreased ER stress as well as subsequent apoptosis. Supplement Figure S5. sI/R led to NRVM apoptosis induced by ER stress but not through the ATF6 and p-IRE1α pathways.
