# Supplementary material for: iPLA2β Contributes to ER Stress-Induced Apoptosis during Myocardial Ischemia/Reperfusion Injury
Source: Cells. 2021 Jun 9;10(6):1446. doi: 10.3390/cells10061446 (PMC8227999; doi:10.3390/cells10061446)
Supplement: Supplementary file 1 [file cells-10-01446-s001.zip › cells-1134195 supplement material.pdf]

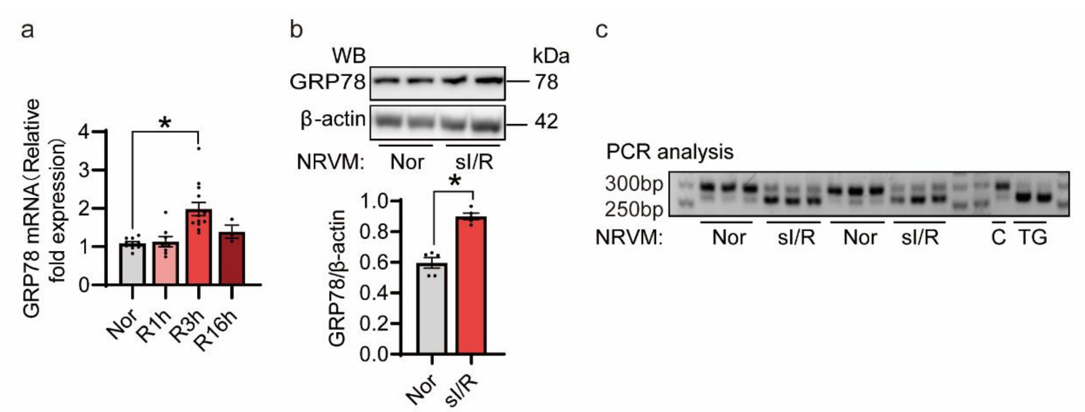

Supplementary Fig S1. Simulated ischemia/reperfusion (sI/R) injury-triggered ER stress in NRVMs. (**a**) sI/R led to strong induction of the ER resident chaperone GRP78 at mRNA level. NRVMs were subjected to ischemia for 3 h, followed by different times of reperfusion. Quantification real-time PCR was conducted to measure mRNA levels with 18S as an internal control. n = 3–11 per group. (**b**) Ischemia for 3 h followed by reperfusion for 3 h induced the expression of the ER chaperone GRP78 at protein level. Immunoblotting showed that GRP78 was significantly increased at protein level. β-actin was used as a loading control. Quantification is shown below. n = 5 per group. (**c**) Effects of sI/R on XBP1 splicing. NRVMs were subjected to 3 h ischemia and 3 h reperfusion or 0.5 μM thapsigargin (TG), an ER stress activator, for 6 h as a positive control. XBP1 splicing: RT-PCR was used to assess the relative levels of unspliced XBP1 (290 bp) and spliced XBP1 (264 bp), as described in the Materials and Methods. Data are represented as mean ± SEM. All experiments were independently replicated in triplicate. *p < 0.05.

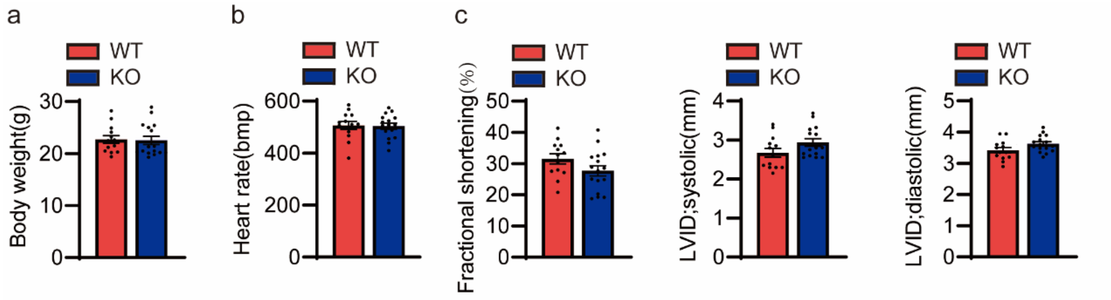

Supplementary Fig S2. Analysis of *Pla2g6*$^{-/-}$ mice and wild-type (WT) mice. (**a**) iPLA$_2$β knockout did not alter the mouse body weight. n = 13-16 per group. (**b**) No changes in heart rate were observed between the *Pla2g6*$^{-/-}$ and wild-type mice. n = 13-16 per group. (**c**) iPLA$_2$β knockout did not affect baseline ventricular contractile function, as evidenced by the lack of significant differences in ventricular fractional shortening, left ventricular internal diameter (LVID) systole, and LVID diastole. n = 13–16 per group. Data are represented as mean ± SEM. All experiments were independently replicated in triplicate. *p < 0.05.

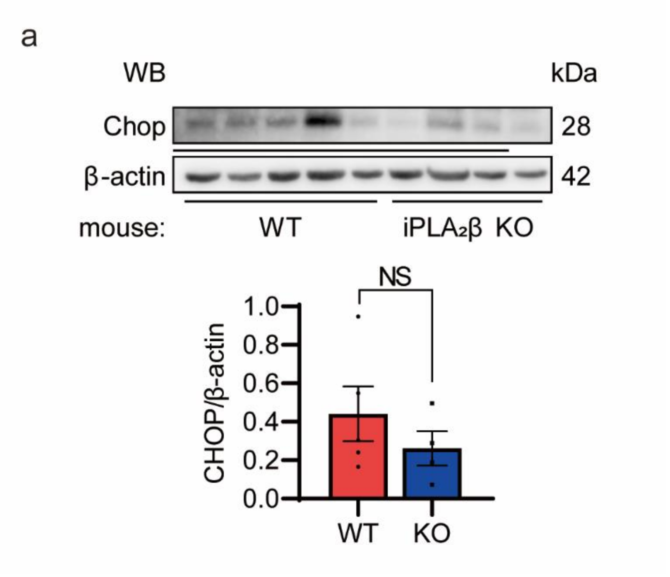

Supplementary Fig S3. No significant difference in the expression of CHOP at basal level between WT and KO mice (**a**) iPLA$_2$β knockout did not affect the expression of basal level of CHOP protein. n = 4–5 per group. Data are represented as mean ± SEM. All experiments were independently replicated in triplicate. *$p < 0.05$.

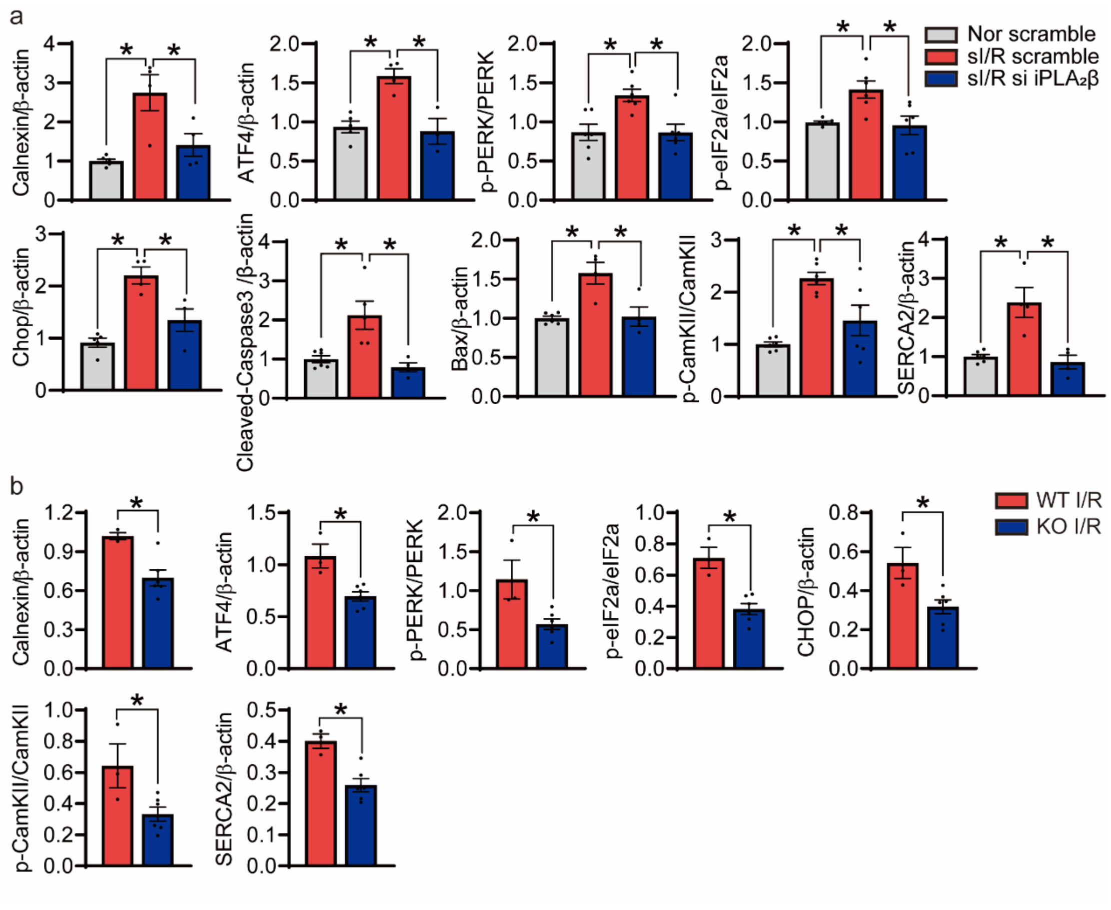

Supplementary Fig S4. The inhibition of iPLA$_2$β *in vitro* and *in vivo* decreased ER stress as well as subsequent apoptosis. (**a**) Upregulated ER stress, apoptotic markers and Ca^{2+} regulate related proteins caused by sI/R decreases with the inhibition of iPLA$_2$β in NRVMs. Quantification is shown. n = 3–6 per group. (**b**) Upregulated ER stress, apoptotic markers and Ca^{2+} regulate related proteins

caused by I/R decreases in *Pla2g6*$^{-/-}$ mice hearts. Quantification is shown. n = 3–6 per group. Data are represented as mean ± SEM. All experiments were independently replicated in triplicate. *p < 0.05.

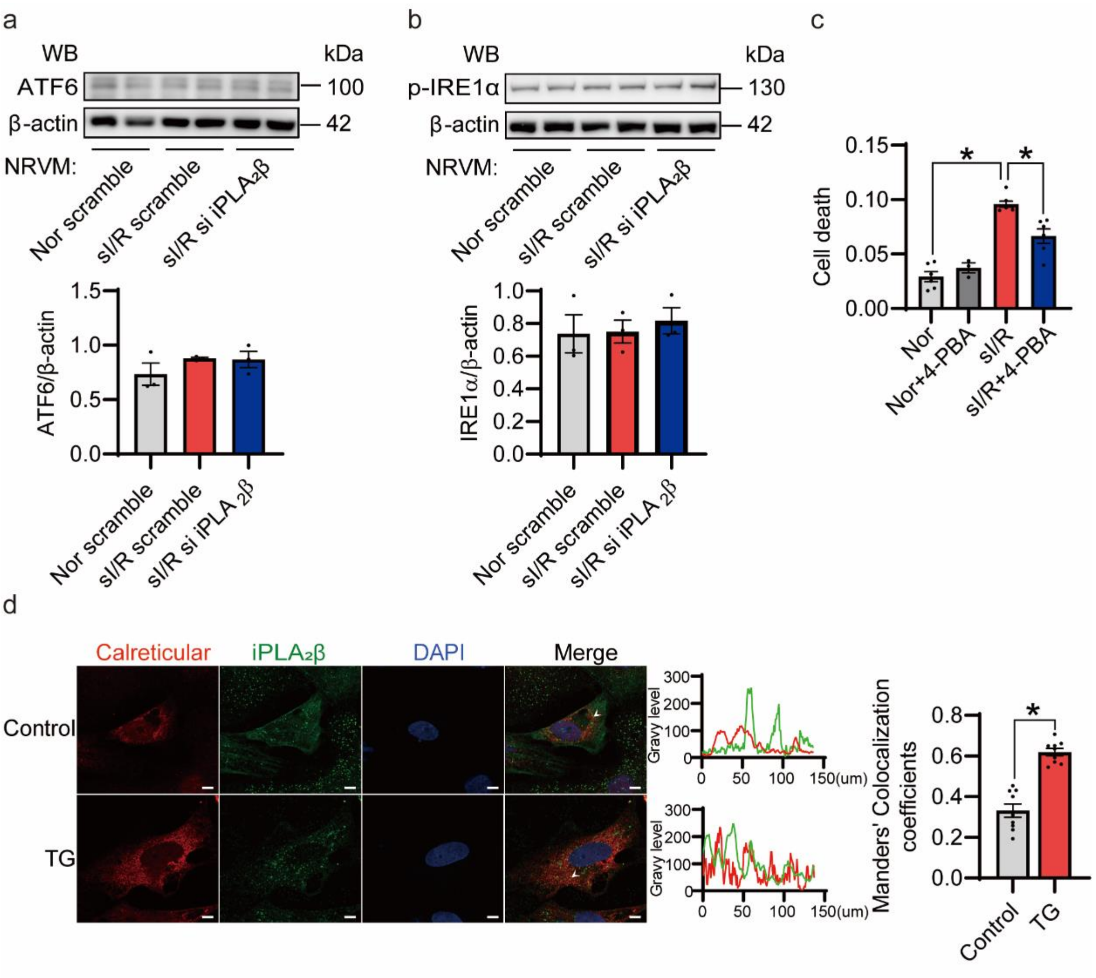

Supplement Fig S5. sI/R led to NRVM apoptosis induced by ER stress but not through the ATF6 and *p*-IRE1α pathways. (**a**) There were no significant differences in the ATF6 protein expression in NRVMs subjected to sI/R. Quantification is shown below. n = 3 per group. (**b**) There were no significant differences in the *p*-IRE1α protein expression in NRVMs subjected to sI/R. Quantification is shown below. n = 3 per group. (**c**) Pretreatment with 5 mM 4-phenylbutyric acid (4-PBA) for 30 min ameliorated myocardial cell death caused by sI/R injury. LDH levels were measured to determine relative cell death. n = 7 per group. (**d**) ER stress caused iPLA$_2$β translocation to ER. NRVMs were treated with 0.5 μM thapsigargin (TG) for 6 h. iPLA$_2$β was revealed by confocal immunofluorescence staining (green). ER was revealed by confocal immunofluorescence staining for calreticulin (red). Scale bar =20 μm. The areas of interest are labeled by white arrows. The signaling intensity for both channels was scanned and recorded on the right. Colocalization of iPLA$_2$β and ER was quantified by Manders' colocalization coefficients (MCCs). n = 7–8 per group. Data are represented as mean ± SEM. All experiments were independently replicated in triplicate. * p < 0.05.
